# Crosstalk between lactic acid and immune regulation and its value in the diagnosis and treatment of liver failure

**DOI:** 10.1515/biol-2022-0636

**Published:** 2023-09-11

**Authors:** Yong Lin, Gengjie Yan, Minggang Wang, Kan Zhang, Faming Shu, Meiyan Liu, Fuli Long, Dewen Mao

**Affiliations:** Graduate School of Guangxi University of Traditional Chinese Medicine, Nanning, Guangxi, China; Department of Hepatology at the First Affiliated Hospital of Guangxi University of Traditional Chinese Medicine, Nanning, Guangxi, China

**Keywords:** liver failure, lactic acid, immunoregulatory response

## Abstract

Liver failure is a common clinical syndrome of severe liver diseases, which belongs to one of the critical medical conditions. Immune response plays a leading role in the pathogenesis of liver failure. Lactic acid as a target for the treatment and prediction of liver failure has not attracted enough attention. Since the emergence of the concept of “histone lactation,” lactic acid has shown great promise in immune response and escape. Therefore, targeted lactic acid may be a reliable agent to solve immune and energy metabolism disorders in liver failure. Based on the relationship between lactic acid and immune response, the cross-talk between lactic acid metabolism, its compounds, and immune regulation and its significance in the diagnosis and treatment of liver failure were expounded in this article to provide new ideas for understanding and treating liver failure.

## 1 Introduction

Liver failure results from the continuous progression or sudden deterioration of various liver diseases. The high medical cost, mortality, and morbidity present a problematic situation of triple superposition, leading to liver failure becoming a major disease requiring comprehensive analysis of complex pathogenesis in the field of liver diseases. Based on the pathophysiological characteristics of liver failure, most studies still point to systemic immune inflammation playing a central role in the process of liver injury and determining the clinical outcome and prognosis [1]. Lactic acid is a metabolic product of pyruvate, which is the end product of glycolysis. Lactic acid not only provides energy for cell growth and development but also acts as an important signaling molecule affecting the biochemical functions of proteins in cells and regulates the biological functions of different kinds of cells [2,3]. Evidence shows that immune cells also consume a large amount of glucose during the immune response [4]. In addition, an immunosuppressive mechanism may be established in the presence of lactic acid to help inflammatory mediators and tumor cells obtain potential immune escape [5,6]. In this sense, lactic acid may act as an intermediary between immune response and immunosuppression [7]. Recently, Zhang et al. proposed a novel epigenetic change mediated by histone lysine lactoacylation [8]. Emerging studies have shown that histone lactation is involved in various cellular events, including immune regulation [2,3,4,8]. Therefore, lactic acid metabolism and its mechanism of action can help in understanding its association with the regulation of immune response during liver failure and act as an effective medium for liver disease diagnosis and treatment, which deserves our further attention.

## 2 Production and metabolism of lactic acid

In living organisms, cells use various metabolic pathways for energy generation and biosynthesis, of which glucose metabolism is the main process. Glucose is converted into pyruvate in the cytoplasm by glycolytic enzymes. At this time, pyruvate is at the crossroads of oxidative phosphorylation (OXPHOS) and fermentation, depending on the aerobic and anaerobic state of the cell [2]. Under aerobic conditions, pyruvate enters the tricarboxylic acid (TCA) cycle in the mitochondria to produce carbon dioxide and water. Each glucose molecule produces 36 adenosine triphosphate (ATP) molecules. In contrast, in the absence of oxygen, lactate dehydrogenase (LDH) reduces pyruvate from cytoplasm to lactic acid, producing only two ATPs [9]. LDH, pyruvate dehydrogenase kinase (PDK), and pyruvate dehydrogenase (PDH) control the conversion of pyruvate. LDH has two main subunits: LDHA and LDHB. Generally, LDHA catalyzes the conversion of pyruvate to lactic acid, while LDHB is responsible for the conversion of lactic acid to pyruvate [10]. The generated lactic acid continues to mediate the activity of PDK phosphorylation of PDH, leading to the obstruction of pyruvate entering the TCA cycle [11]. On the one hand, it reduces glucose consumption through OXPHOS; on the other hand, it causes pyruvate accumulation and indirectly promotes lactic acid production. In addition, the shuttle movement of lactic acid between cells and the microenvironment mainly depends on the monocarboxylic acid transporter (MCT) system, in which the inflow and outflow depend on MCT1 and MCT4, respectively [12]. When lactic acid is transferred between cells, it disrupts the pH homeostasis outside the cell, resulting in an acidic environment that affects enzyme activity and the regulation of immune cells. Consistent with this, energy metabolism preferentially switches from OXPHOS to glycolysis when the liver is damaged, resulting in the partial conversion of pyruvate to lactic acid [13]. From this perspective, the level of lactic acid is correlated with the degree of liver damage, and the production of lactic acid is closely related to the immune regulatory response of the liver.

## 3 Relationship between lactic acid and immune regulation in the liver

### 3.1 Nonspecific immunity

#### 3.1.1 Dendritic cells (DCs)

DC is the strongest antigen-presenting cell in the human body, residing in the central vein and portal region of the liver. After antigen uptake, DC differentiates and matures, migrates to secondary lymphoid tissue, interacts with T cells initially, and initiates acquired immunity [14]. There are three subtypes of DCs in the liver: myeloid (MDCs), lymphoid (LDCs), and pre-plasma-cytoid DCs (pDCs). When the liver receives stimulation, glycolysis is enhanced, and a large amount of lactic acid is secreted within a few minutes after DC activation [15]. Generally, MDCs secrete IL-12 and induce Th1 cells and cytotoxic T lymphocyte (CTL) immune response, but studies have found that the increase in exogenous lactic acid can inhibit the differentiation and maturation of MDCs [16]. LDCs induce a Th2 immune response. When stimulated by foreign antigens, LDCs and pDCs produce a large amount of type I interferons, which directly inhibit viral replication and activate natural killer (NK) cells, B cells, T cells, and MDCs to induce and enhance antiviral immune response. Lactate can interact with the lactate receptor G-protein-coupled receptor 81 on pDCs to inhibit antigen presentation by interfering with antigen degradation in the endosomes [17]. In addition, lactate enhances tryptophan metabolism and kynurenine production by pDCs, which contribute to the induction of FoxP3 CD4 Tregs [18]. Furthermore, the liver immunomodulatory analysis showed that during liver inflammation or in solid tumors of the liver, the continuous increase in lactic acid transformation results in high lactate levels in cells, while DCs in an acidic environment are characterized by reduced glucose consumption, increased lactate output, and upregulated mitochondrial oxidative metabolism [19], and the combined action of the two inevitably interferes with immune initiation and response.

#### 3.1.2 NK and natural killer T (NKT) cells

In the liver, the NK cells, unlike T and B cells, can directly produce a target cell-killing effect without specific antigen stimulation and secrete many inflammatory factors, such as IFN-γ, TNF-α, and IL-3, which play a vital role in antiviral and immune regulation of the body [20]. NK cells can be divided into two types based on the expression of transcription factors, namely circulating conventional NK cells in the blood and tissue-resident NK cells (trNK) [21]. Studies have shown that LDHA-mediated aerobic glycolysis and OXPHOS are essential for NK cell proliferation and maintenance of its antiviral and antitumor capabilities [22,23]. For example, in tumors or virus-carrying liver, impaired mitochondrial function mediated by elevated lactate levels leads to early apoptosis of trNK cells [21]. However, in the cellular microenvironment, the decrease in the pH value of tumor cells further amplifies the lactic acid-inhibited immune response [24] because the tumor-derived lactic acid downregulates the expression of the activated NK receptors NKp46 and NKG2D. This results in the decreased expression of perforin and granase [25,26], thus reducing the antiviral activity of NK cells. Conversely, blocking the flow of lactic acid into NK cells or increasing the oxygen level in the microenvironment can restore or enhance the toxic function of NK cells and cytokine production [27,28]. NKT cells originate from the thymus, express receptors on the surface of T cells and NK cells, and are activated by recognizing the antigen presented by the MHC molecule CD1d to release several cytokines such as IFN-γ and IL-4, regulate the balance between Th1 and Th2, and participate in important processes such as immune diseases and antitumor and antiviral activities in the liver. Studies have shown that NKT cells depend more on OXPHOS to proliferate and promote cytokine expression. However, high levels of extracellular lactic acid block glycolysis in NKT cells, negatively affecting their survival and functional expression [29].

#### 3.1.3 Macrophage

In liver immunity, macrophages mainly express surface molecules related to antigen uptake, including complement receptors, scavenger receptors, and toll-like receptors, and take up exogenous antigens and present them to activated effector T cells, enhancing T cell biological activity. Macrophage polarization can be mainly divided into two types: classically activated macrophages (M1 macrophages) and alternately activated macrophages (M2 macrophages) [30]. M1 macrophages are highly effective effector cells with anti-inflammatory and immune regulation effects, while M2 macrophages are involved in pro-inflammatory processes, adaptive Th1 immunity, tissue remodeling and repair, and tumor progression [31]. Studies have shown that LDHA plays a key role in regulating macrophage polarization. The loss of LDHA in macrophages promotes polarization of M1-like macrophages, resulting in decreased expression of vascular endothelial growth factor (VEGF) and increased activity of effector CD8^+^ T cells [32]. On the contrary, LDHA promotes the polarization of M2 macrophages by promoting the expression of hypoxia-inducing factor HIF-1a, increasing the expression of VEGF [33]. So far, macrophages have been found to facilitate the M1-to-M2 and M2-to-M1 phenotype transitions. However, a recent study showed that tumor acidosis induces the phenotypic transformation of regulatory macrophages, which promotes tumor growth. The specific mechanism involves the activation of plasma membrane G-protein-coupled receptors mediated by acidosis and downstream cyclic adenosine phosphate signaling. This blocks the M2 gene transcription and thus inhibits the expression of inflammatory genes TNF and Nos2 [34], suggesting that lactic acid may mediate partial cross-talk between tumor cells and macrophages. From similar conclusions, it can be inferred that lactic acid promotes the expression of inflammatory bodies and pro-inflammatory factors such as IL-1β, IL-10, IL-6, and HIF-1α by downregulating the phosphorylation of p65-NFκB in macrophages or enhancing TLR4 signaling in macrophages [35,36]. Although the specific cross-talk mechanism between lactic acid and macrophages is still unclear, it is certain that long-term exposure of cells to lactic acid leads to mitochondrial autophagy in macrophages, causing effects such as decreased mitochondrial function, increased reactive oxygen species, and impaired oxidative ATP production, which damage the function of macrophages [37].

### 3.2 Specific immunity

#### 3.2.1 CD4^+^ T cells

CD4^+^ T cells include Th1, Th2, CD4^+^ Treg, and Th17 cells. When T cells are activated, aerobic glycolysis converts glucose into lactic acid to meet energy and biosynthesis requirements [38]. Evidence has shown that lactic acid is directly or indirectly involved in CD4^+^ T cell expression. Intuitively, lactic acid regulates CD4^+^ T cell polarization and reduces the percentage of anti-tumor Th1 subpopulation by inducing silencing regulatory protein silent information regulator sirtuin 1 (SIRT1)-mediated T-box expressed in T cells (T-bet) transcription factor deacetylation [39]. Indirectly, LDHA is a key enzyme converting pyruvate into lactic acid. It regulates the expression of IFN-γ in Th1 cells by regulating the acetylation of histone H3 at lysine 9 and lysine 3 (H3K3me3) (Th1 cells are mainly involved in the immune response by secreting IL-2 and IFN-γ) [40]. In addition, lactic acid directly inhibits the movement of T cells in inflammatory tissues, leading to the trapping of T cells and the production of pro-inflammatory cytokines to amplify immune inflammatory response [41]. This may be attributed to the fact that when the extracellular lactic acid level is high, lactic acid enters CD4^+^ T cells through MCT1 and converts into pyruvate through LDHB, resulting in the downregulation of T cell glycolysis, blocking the output of lactic acid by T cells, and the accumulated lactic acid disrupts CTL metabolism [42]. Similarly, a lactate-rich environment interferes with the glycolysis of CD4^+^ T cells through the lactate transporter SLC5A12 and reduces their chemotaxis to the chemokine CXCL10, thus preferentially differentiating Th17 cells and inflammatory subsets [43].

The major subtype of Treg, CD4^+^ CD25^+^ Treg, is a group of T cells that specifically express the transcription factor Foxp3, and its ratio to effector T cells plays an important role in immunosuppression and maintenance of immune balance. The lactic acid in the tumor microenvironment helps Tregs to proliferate and maintain their immunosuppressive function [44]. This can be because lactic acid is transported to the initial CD4^+^ T cells via MCT1, which activates the expression of NF-κB and Foxp3 (NF-κB is a key regulatory factor of Foxp3 transcription). The high expression of Foxp3 upregulates the proportion of Tregs by inhibiting glycolysis and enhancing OXPHOS, leading to the decline in anti-tumor immunity. On the contrary, high glucose or low lactate levels can inhibit its function and stability [45]. In addition, Tregs use lactic acid to promote the entry of the nuclear factor of activated T cell 1 into the nucleus and induce the expression of programmed death receptor 1, thus making Treg cells more adaptable to low glucose and high lactic acid levels [46].

#### 3.2.2 CD8^+^ T cells

**Figure 1:**
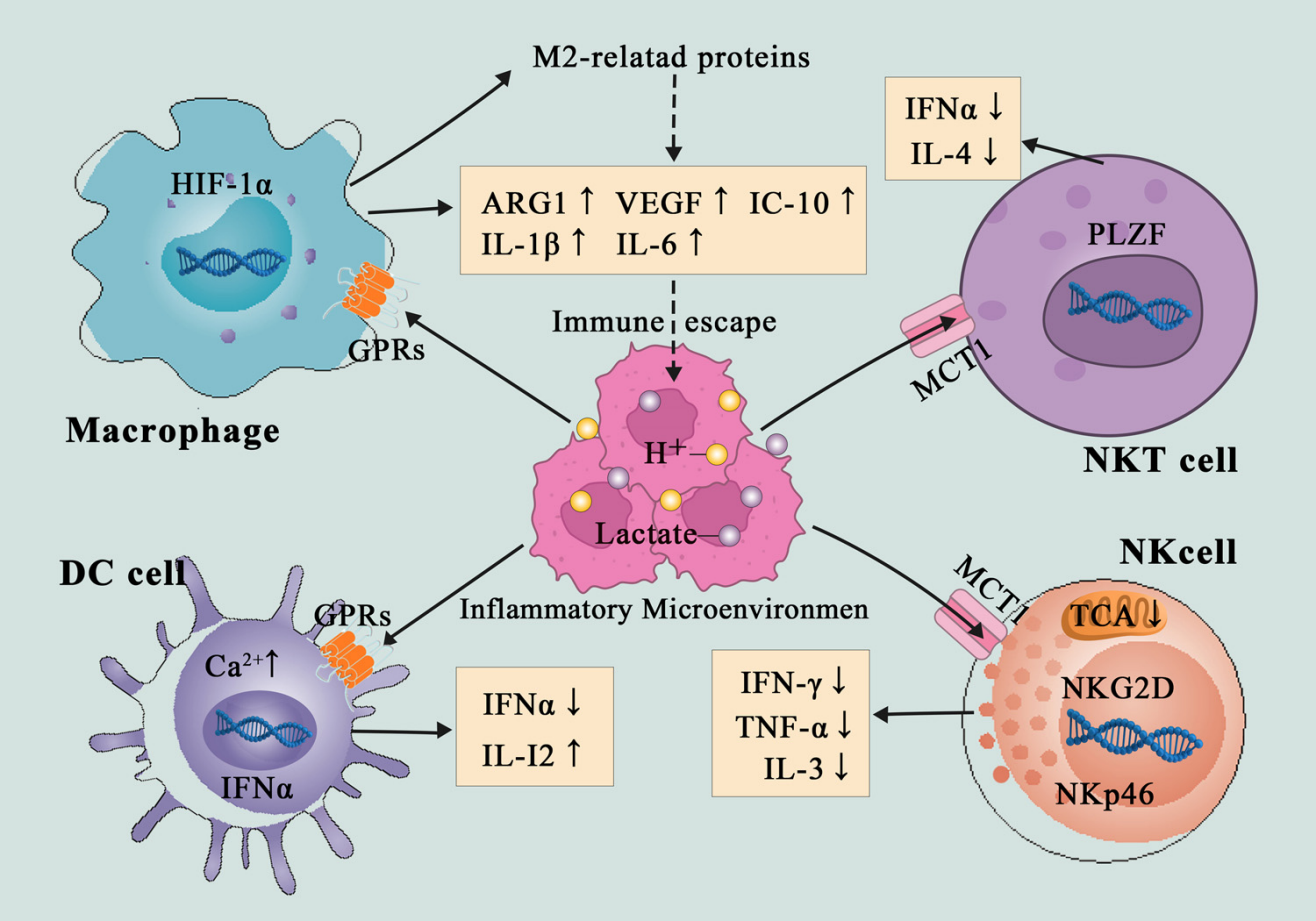
Effect of lactic acid on immune cells in an inflammatory microenvironment. Lactate induces immunosuppression, acidosis, and immune escape by regulating the expression of various genes.

CD8^+^ T cells primarily recognize antigen polypeptides presented by MHC Class I molecules. Similar to CD4^+^ T cells, LDHA deficiency reduces the antitumor activity of CD8^+^ T cells and may also prevent their movement and proliferation [47]. High levels of glycolytic enzymes are also critical for CD8^+^ T cells to express IFN-γ during an immune response [48]. In contrast to the T-reg response, lactic acid from tumor cells inhibits NFAT expression in CD8^+^ T cells, thus reducing the production of IFN-γ and maintaining immune balance with T-reg cells. However, excess lactic acid in tumor cells leads to immune tolerance, and neutralization with proton pump inhibitors can restore the T cell function [49]. The specific mechanism may be that a high lactate level damages the c-Jun N-terminal kinase pathway and the phosphorylation of p38 protein triggered by T cell receptors, thus inhibiting the function of CTL [50] (Figure 1).

## 4 Lactic acid is closely related to liver failure

**Figure 2:**
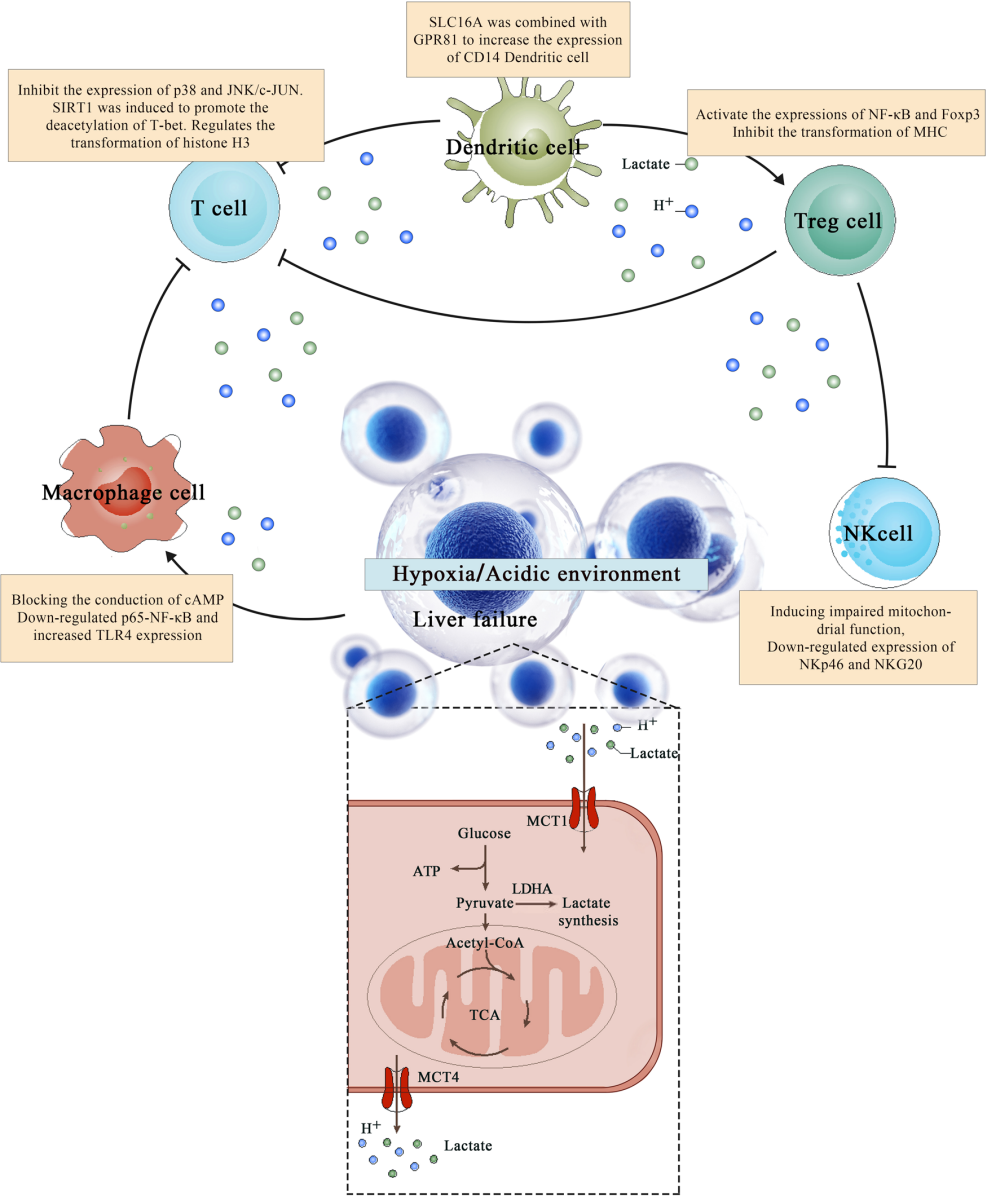
Diagram showing crosstalk between lactic acid and immune regulation. The crosstalk mechanism between lactic acid and various immune cells is shown in the box.

Acute injury during liver failure leads to the activation of innate immune cells, triggering a cascade of cytokines and chemokines, followed by an aggressive systemic inflammatory response syndrome, which is the overall characteristic of immune disorders in liver failure. There are many interactions between host innate immune activation and adaptive immune response to control the overall immune outcome of liver failure. From the above discussion, we know that lactic acid synthesis and secretion are embodied in the whole process of the immune response during liver failure (Figure 2). The effect of lactate levels on liver failure was described in the Lancet in 2002 [51]. Cohort studies have shown that arterial lactate levels are significantly higher in patients with acute liver failure (ALF), and their levels are more rapid and accurate in identifying the outcome of patients with paracetamol-induced ALF [51]. Subsequent studies have been more broadly interpreted [52,53,54,55]. For example, early lactate value is a strong marker of post-hepatectomy liver failure and has the potential to guide postoperative care [52]. The combination of NK cell frequency and lactate levels on admission can reliably predict the survival rate of ALF patients [53]. However, a cohort study found that the LDH level had a prognostic value similar to the end-stage liver disease model (MELD), and the combined prediction method was superior to the two considered separately [54]. Interestingly, the combination of lactic acid levels with the chronic liver failure-sequential organ failure assessment score (CLIF-SOFA) for sequential organ failure also significantly improved the prediction of short-term prognosis in patients with HBV-acute-on-chronic liver failure compared with the use of only CLIF-SOFA [55]. Moreover, during severe complications associated with liver failure (ketoacidosis, hepatic encephalopathy, and sepsis), lactic acid elevation can be an independent risk factor [56,57,58]. Furthermore, lactic acid prediction is applicable in hepatic encephalopathy and high intracranial pressure associated with liver failure. An elevated lactate/pyruvate ratio indicates that the accumulation of glutamine impairs mitochondrial function and leads to intracranial hypertension [59], and lactate is involved in the physiological and pathological reactions of liver failure from the early stage [60]. In contrast, pyruvate kinase deficiency can lead to severe liver dysfunction [61]. In addition, enzymes and compounds related to lactate synthesis and metabolism have shown similar values in animal research as clinical findings. The translocation of PDH and LDH to the nucleus of liver failure mice resulted in increased nuclear concentrations of acetyl-CoA and lactic acid and led to the expression of histone H3 hyperacetylation and damage response genes. Inhibitors of the two enzymes can reduce liver injury and improve survival rates [62]. Ethyl pyruvate (EP) can reduce intestinal permeability, inhibit a variety of pro-inflammatory cytokines in ALF rats, and protect rat liver function [63]. Recently, Zhou et al. [64] determined that contrary to their typical role as anti-inflammatory agents in the host, indole-3-acetic acid (IAA) and indole-3-lactic acid (ILA) gavage-sensitized mice to D-GalN/LPS-induced-ALF with a rapid increase in serum transaminases and histologic lesion. This can be attributed to the exacerbation of D-GalN/LPS-induced ALF via the probable involvement of the Tlr2/NF-κB pathway and ileac dysbiosis characterized by enriched Gram-positive genus due to the IAA pretreatment. In addition, Gan et al. [65] found that the acidic microenvironment caused by elevated lactic acid could inhibit the production and function of CD25, CD3, Foxp3, and iTregs through the PI4K/Akt/mTOR signaling pathway, leading to liver dysfunction. In contrast, the reversal of the acidic microenvironment restored Foxp3 expression and iTreg function. Moreover, the proton pump inhibitor omeprazole improved the decreased iTreg differentiation caused by the acidic microenvironment.

## 5 Summary

The physiological and pathological characteristics of lactic acid play a wide range of roles in immune regulatory responses. It is certain that elevated lactate levels not only inhibit the anti-inflammatory and anti-tumor effects of immune cells such as CD4^+^ T cells, CD8^+^ T cells, NK cells, and NKT cells but also benefit immunosuppressive cells such as Treg cells. The physiological vector response of lactic acid can maintain the proportion of effector T cells and maintain immune balance. Pathologically, with the increase in lactic acid levels, the energy metabolism cycle, mainly glycolysis, is seriously affected, and the immune balance is immediately destroyed, and even immune escape and immunosuppression occur. During liver failure, the metabolism of lactic acid may be closely related to the continuous disorder and sudden outbreak of immune inflammatory response, and the diagnosis and treatment of liver failure targeting lactic acid, such as LDHA and EP, has shown great developmental prospects. However, lactic acid as a therapeutic strategy still faces many challenges. For example, the starting point of lactic acid and immune regulation is the relationship between immune cells and glucose metabolites; whether this relationship involves energy supply, redox action, glucose conversion, glycolytic metabolism, and many other physiological links is yet to be explored. At the same time, it is worth noting that lactic acid and immune cells are also associated with several low molecular compounds, such as caffeine [66], solute carrier transporters [67], and tryptophan (including ILA, indole-3-acrylate, and indole-3-propionic acid) [68]. Therefore, the relation between liver failure and other liver diseases and lactic acid and lactate needs to be further explored, and this concept can help improve the diagnosis and treatment strategy for liver failure in the future.
